# Alpha-1 Antitrypsin Z Variant (AAT PI*Z) as a Risk Factor for Intrahepatic Cholestasis of Pregnancy

**DOI:** 10.3389/fgene.2021.720465

**Published:** 2021-09-07

**Authors:** Przemyslaw Kosinski, Malgorzata Kedzia, Adrianna Mostowska, Pawel Gutaj, Michal Lipa, Ewa Wender-Ozegowska, Adriana Rozy, Joanna Chorostowska-Wynimko, Miroslaw Wielgos, Aleksandra Jezela-Stanek

**Affiliations:** ^1^First Department of Obstetrics and Gynecology, Medical University of Warsaw, Warsaw, Poland; ^2^“Club 35”, Scientific Group of Polish Society of Obstetricians and Gynaecologists, Warsaw, Poland; ^3^Division of Reproduction, Department of Obstetrics, Gynecology, and Gynecologic Oncology, Poznan University of Medical Sciences, Poznań, Poland; ^4^Department of Biochemistry and Molecular Biology, Poznan University of Medical Sciences, Poznań, Poland; ^5^Department of Genetics and Clinical Immunology, National Institute of Tuberculosis and Lung Diseases, Warsaw, Poland

**Keywords:** ursodeoxycholic acid, alpha-1 antitrypsin deficiency, intrahepatic cholestasis of pregnancy, cholestasis, pregnancy

## Abstract

**Background:** Intrahepatic cholestasis of pregnancy (ICP; prevalence 0.2–15.6%) is the most common pregnancy-related liver disorder. It may have serious consequences for a pregnancy, including increased risk of preterm delivery, meconium staining of amniotic fluid, fetal bradycardia, distress, and fetal demise. In cases of high bile acids (>100μmol/L), patients have 10-fold increase in the risk of stillbirth. Biophysical methods of fetal monitoring, such as cardiotocography, ultrasonography, or Doppler have been proven unreliable for risk prediction in the course of intrahepatic cholestasis. Therefore, we believe extensive research for more specific, especially early, markers should be carried out. By analogy with cholestasis in children with inherited alpha-1 antitrypsin deficiency (AATD), we hypothesized the *SERPINA1* Z pathogenic variant might be related to a higher risk of cholestasis in pregnancy. This study aimed to investigate the most common AATD variants (Z and S SERPINA1 alleles) in a group of cholestatic pregnant women.

**Results:** The Z carrier frequency was calculated to be 6.8%, which is much higher compared to the general population [2.3%; the Chi-squared test with Yates correction is 6.8774 (*p*=0.008)].

**Conclusion:** Increased prevalence of *SERPINA1* PI*Z variant in a group of women with intrahepatic cholestasis may suggest a possible genetic origin of a higher risk of intrahepatic cholestasis in pregnancy.

## Introduction

The *SERPINA1* (Serpin Peptidase Inhibitor, Clade A, Member 1, MIM *107400) gene encodes alpha-1 antitrypsin [AAT, A1AT, formerly known as a protease inhibitor (PI)], a major plasma serine PI (Ferrarotti et al., 2012). The *SERPINA1* gene is highly polymorphic, with more than 100 clinically significant variants. The most prevalent pathogenic alleles are PI*Z (c.1096G>A, p.Glu366Lys) and PI*S (c.863A>T, p.Glu288Val) identified in 95% of severe alpha-1 antitrypsin deficient (AATD) patients (McElvaney et al., 1997). Both result in a quantitative and functional alpha-1 antitrypsin deficiency (AATD). The serum levels of alpha-1-antitrypsin have also been recently found to change during pregnancy according to differential DNA methylation of *SERPINA1* gene (Rotondo et al., 2020). It underlines that AATD might also be potentially due to epigenetic modifications other than to *SERPINA1* variants. AATD in adults is characterized by a variety of clinical presentations (Stoller et al., 1993), while in children, the most common manifestation is neonatal cholestasis, also called “cholestatic hepatitis.”

Intrahepatic cholestasis of pregnancy (ICP) is the most common pregnancy-related liver disorder with incidence of 0.1–1.5% in Central/Western Europe and North America and up to 1.5–4% in Chile and Bolivia (Brouwers et al., 2015). ICP usually ensues in the second or third trimester and spontaneously withdraws within 2–3weeks post-delivery. It commonly reoccurs in subsequent pregnancies (45–70%; Lee et al., 2008). The pathomechanism of ICP is not fully understood. It is thought to be multifactorial, with both environmental, hormonal as well as genetic factors involved. As ICP may have serious consequences for the course of pregnancy, including increased risk of preterm delivery, meconium staining of amniotic fluid, fetal bradycardia, distress, and fetal demise (Rook et al., 2012), it may be clinically beneficial to search for additional diagnostic markers or predictors that would allow earlier identification of patients at higher risk of ICP. Given the link between the deficient variants of the *SERPINA1* gene and pediatric intrahepatic cholestasis as a manifestation of AATD (Chen et al., 2018; Comba et al., 2018; Lin et al., 2019), we aimed to assess the presence and frequency of the two most common *SERPINA1* nucleotide variants – PI*Z and PI*S – in a group of 103 women with a history of ICP.

## Materials and Methods

The study group consisted of 103 pregnant females with ICP diagnosed according to current guidelines (Manzotti et al., 2019), i.e., peak serum bile acid concentration above 10μmol/L. The characteristics of the study group are presented in Table 1. All pregnant women were otherwise healthy and in particular co-morbidities such as non-alcoholic fatty liver disease, cystic fibrosis, COPD, or alcohol abuse were excluded. The average gestational age (GA) at the time of occurance of cholestasis was 33weeks. After the diagnosis, all patients were treated with ursodeoxycholic acid (UDCA) starting with a dose of 250mg three times a day. During the treatment the patients were hospitalized and fetal and maternal well-being were monitored. The UDCA dose was modified by experienced physician according to the therapeutic effect and intensified in case of progressive increase of total bile acids (TBA). The maximal applied dose did not exceed 1,500mg/day. UDCA was administered until the day of delivery. The TBA and aminotransferases concentrations were monitored after fasting period regularly: twice a week or even daily in selected cases. Written informed consent was obtained from all participating individuals. The study procedure was approved by the Local Ethics Committees of Poznan University of Medical Sciences (no. 197/18) and performed in accordance with the code of ethics of the Declaration of Helsinki.

**Table 1:** Characteristics of patients with cholestasis of pregnancy [the data is presented as median (range)].

Number of patients (*n*)	103
Age (years)	30 (21–46)
Parity	1 (1–6)
Gestational age at diagnosis	33 (13–39)

## Methods

### Genotyping of PI*S and PI*Z AAT Alleles by Real-Time PCR

Genomic DNA was isolated from peripheral blood lymphocytes with the salting-out method. In this DNA extraction technique initially described by Miller et al. (1988), following cell lysis and proteinase K treatment, the cell debris and proteins are precipitated using a high-concentration salt solution. The DNA is then precipitated using ethanol and redissolved in sterile H_2_O. The quality and quantity of DNA were checked spectrophotometrically (*NanoDrop One* spectrophotometer, Thermo Fisher Scientific, United States). AAT genotyping was performed in the LightCycler 480 II machine (Roche Diagnostics Ltd., Switzerland) using a set of specific oligonucleotide primers and minor groove binding (MGB) hydrolysis probes for PI*S and PI*Z alleles (sequences available upon request; Struniawski et al., 2013). Each assay included an S/Z heterozygote, Z/Z homozygote. Two duplex real-time PCR reactions were performed simultaneously using fluorescent hydrolysis probes for the detection of both wild-type and variant alleles. The assembled reaction mixtures were amplified in 96-well microplates (Roche Applied Science) using the following cycling conditions: 10min incubation at 95°C followed by 40cycles at 95°C for 20s, and at 60°C for 60s. Fluorescence emissions of PI*S and PI*Z probes were detected in the FAM channel and VIC channel, respectively, during the 60°C annealing step of each PCR cycle. The amplification results were interpreted according to the conventional endpoint genotyping principles using the LightCycler 480 Software, version 1.5 (Roche Applied Science). A two-tailed Chi-squared test was employed to evaluate significance of difference in the rate of the *SERPINA1* Z pathogenic variant between studied group and general population (Chorostowska-Wynimko et al., submitted manuscript). A *post hoc* analysis revealed that for the observed difference in the frequency in the rate of the *SERPINA1* Z pathogenic variant between studied group and general population our study had 74.1% power (alpha 0.05) to detect significant difference.

## Results

Final results were obtained from 103 DNA samples. The *SERPINA1* variants of interest were detected in seven (PI*Z, 6.8%) and 1 (PI*S, 0.9%) patients with ICP. All identified individuals were heterozygous, PI*MZ or PI*MS, respectively. Their demographic and clinical details are presented in Table 2A. Clinical characteristics of neonates born to PI*MZ and PI*MS heterozygous mothers are presented in Table 2B. The Chi-squared test for the rate of the *SERPINA1* Z pathogenic variant between studied group and general population with Yates correction is 6.8774 (*p*=0.008).

**Table 2A:** Demographic and clinical characteristics of PI*MZ heterozygotes (*n*=7) and PI*MS (*n*=1, highlighted in italics) identified in the group of 103 patients with intrahepatic cholestasis of pregnancy (ICP; premature births marked in bold).

	CH5	CH41	**CH53**	CH68	**CH75**	**CH95**	K_202	*CH46*
Age (years)	33	30	**28**	37	**38**	**29**	29	35
Obstetric history^*^	1–0-0	1–0–0	**1–0–0**	2–0–1	**1–0–2**	**0–0–0**	1–0–0	*1–0–0*
History of cholestasis	−	−	**−**	−	**−**	**−**	−	*+*
WOG at diagnosis	33	38	**31**	33	**28**	**30**	30	*36*
Treatment	+	+	**+**	+	**+**	**+**	+	*+*
Symptoms	+	+	**+**	+	**+**	**+**	+	*+*
Family history	−	−	**+**	−	**+**	**−**	+	*−*
ALT U/L	66	90.1	**36**	166.7	**275.9**	**166.7**	84.1	*30.0*
AST U/L	137.6	113	**52.3**	246.9	**333.5**	**88.5**	181.3	*27.6*
TBA (μmol/L)	35.5	32.1	**171.3**	44.3	**59**	**39.5**	259	*16.1*
GA at delivery (weeks)	37	38	**33**	37	**32**	**36**	38	*38*
Delivery	Vaginal	Vaginal	**CS**	Vaginal	**CS**	**Vaginal**	CS	*CS*
Induction	+	−	**Iatrogenic**	+	**−**	**+**	−	*−*

WOG, week of gestation; GA, gestational age; ALT, alanine aminotransferase; AST, aspartate aminotransferase; TBA, total bile acids; and CS, cesarean section.

* Includes: at term delivery-preterm delivery-miscarriage. “+” means YES. “-” means NO.

**Table 2B:** Clinical characteristics of neonates born to PI*MZ and PI*MS (highlighted in italics) heterozygous mothers with recent history of ICP (see Table 2A).

	CH5	CH41	CH53	CH68	CH75	CH95	K_202	CH87	*CH46*
Amniotic fluid	Clear	Clear	Clear	Clear	Clear	Clear	Clear	Clear	*Clear*
Weight (g)	3,320	2,820	2,140	3,100	1,600	3,100	3,080	3,290	*3,020*
1'Apgar score	10	10	9	10	9	10	10	8	*10*
5'Apgar score	10	10	10	10	8	10	10	9	*10*
pH-arterial	7.34	7.28	7.31	7.35	7.32	7.30	7.29	7.28	*7.33*
Need for ventilation	−	−	+	−	+	−	−	−	*−*
NICU admission	−	−	+	−	+	−	−	−	*−*
Days in hospital	5	5	17	6	24	3	3	3	*3*
Hyperbilirubinemia	+	+	+	+	+	−	−	−	*−*
Phototherapy	−	−	+	−	+	−	−	−	*−*

No stillbirths and no newborn deaths occurred at any of the patients with cholestasis in the examined group. “+” means YES. “-” means NO.

After completed the study, we have retrospectively analyzed the medical files of women diagnosed as alpha-1 antitrysin deficiency carriers. No abnormalities suggesting liver dysfunctions [i.e., increased alanine aminotransferase (ALT), aspartate aminotransferase (AST), or bilirubin] were noted in any individuals. Family histories were also non-remarkable as far as lung diseases are concerned.

A detailed health assessment is planned, as well as further monitoring in regards to liver dysfunctions. All women will receive genetic counseling to inform of the potential risks, management and identification of indications for genetic testing toward AAT deficiency/carriership in close family members (siblings and offspring).

## Discussion

Intrahepatic cholestasis of pregnancy is associated with an increased risk of fetal morbidity and mortality. Elevated TBA level, resulting in ICP, is also a risk factor for an adverse perinatal outcome, including spontaneous preterm birth, meconium-stained amniotic fluid, and neonatal unit admission (Glantz et al., 2008; Herrera et al., 2018; Chappell et al., 2019); Based on recent meta-analyses, the risk of stillbirth is increased in women with ICP and singleton pregnancies when serum bile acids concentrations are of 100μmol/L or more (Ovadia et al., 2019). Also other researchers confirmed that patients with serum bile acids >100μmol/L present an approximately 10-fold higher probability of stillbirth (Kawakita et al., 2015; Ovadia et al., 2019). The diagnosis is based on clinical manifestations, mainly pruritus, as well as abnormal TBA levels (Manzotti et al., 2019). Commonly available biophysical methods of fetal monitoring, such as cardiotocography, ultrasonography, or Doppler ultrasonography, have been proven unreliable for stillbirth risk assessment (Oztas et al., 2015). Therefore, identification of high-risk group for ICP would be of great value in the scientific and potentially also in the clinical setting. As for instance functional genetic variants are considered as biomarkers in oncology, our findings may help to establish new biomarkers for ICP. By analogy with cholestasis observed in children with inherited AATD, we hypothesized the *SERPINA1* PI*Z deficiency variant might be linked to a higher risk of cholestasis in pregnancy.

According to the Online Mendelian Inheritance in Man’s “Gene-Phenotype Relationships,” certain variants of *SERPINA1* gene are associated with: (1) Emphysema due to AAT deficiency (**#** 613490); (2) Emphysema-cirrhosis, due to AAT deficiency (**#** 613490); and (3) Hemorrhagic diathesis due to antithrombin Pittsburgh (**#** 613490; https://www.omim.org/entry/107400, accessed 10, 2020). The clinical manifestations observed in severe inherited AATD are, however, more diverse, and apart from those listed in Table 3, include bronchial asthma, bronchiectasis, panniculitis, and granulomatosis with polyangiitis (GPA; Stoller et al., 1993).

**Table 3:** *SERPINA1* clinical synopses (adapted from OMIM).

Number	# 613490
Title	ALPHA-1 ANTITRYPSIN DEFICIENCY; A1ATD
Inheritance	Autosomal recessive
Respiratory	Dyspnea (onset >35years in smokers, >45years in nonsmokers)
	*Airways* Small airways
	*Lung* Alveolar wall destruction Emphysema, predominantly basal pattern Chronic obstructive pulmonary disease
Liver	Abnormal liver function tests Hepatic intracellular inclusions in ZZ homozygotes Infantile liver abnormalities in <20% with AAT deficiency Cirrhosis (rare)
Neoplasia	Increased hepatocellular carcinoma risk
Laboratory abnormalities	Serum alpha-1-antitrypsin (Pi) deficiency Abnormal liver function tests (AST and ALT)
Miscellaneous	PI*Z allele most common Secondary prevention, avoid smoking, alcohol, and oxidants

In children, neonatal cholestasis is the most common presentation of severe AAT deficiency resulting from PI*ZZ genotypes. It affects ~10% of newborns and infants and was first mentioned by Aagenaes et al. (1972), who document five individuals (Bouchecareilh, 2020). There are more than 50 known pathogenic variants of the *SERPINA1* gene (ClinVar https://www.ncbi.nlm.nih.gov/clinvar, accessed 10, 2020). The PI*Z, a glutamate-to-lysine substitution at position 342, and the PI*S, a glutamate-to-valine substitution at position 264, are considered the most clinically *SERPINA1* variants (Schneider et al., 2020). The frequency of *SERPINA1* Z and S alleles in the Polish population was preliminarily established in a newborn population screening of 658 subjects performed using dry blood spots (DBS) collected in Warsaw between September and December 2011 (Chorostowska-Wynimko et al., 2013). Main deficiency variants were detected in 28 individuals – the PI*Z allele in 18 (2.7%) and the PI*S allele in 10 (1.5%). More recently, in a group of 4,185 unselected neonates, the pathogenic variants were identified in 186 newborns, specifically the PI*Z allele in 96 and the PI*S allele in 90 individuals, corresponding to the estimated prevalence of accordingly 2.3 and 2.1% in the Polish population (Chorostowska-Wynimko et al., submitted manuscript). Meanwhile, the calculated frequency of the PI*Z variant in the ICP was 6.8%, thus considerably higher than expected for the general population. The obvious gender imbalance in the study group is of no clinical importance, as frequencies of *SERPINA1* pathogenic variants are similar in both sexes.

The risk of cholestasis in pregnancy has been associated with genetic variants in *ABCB4*, *ABCB11*, *ATP8B1*, *ABCC2*, and *TJP2* genes (Chen et al., 2018). Heterozygotes mutation in the *ABCB4* (ATP-Binding Cassette, Subfamily B, Member 4) gene on chromosome 7q21 accounts for about 15% of ICP cases (Ziol et al., 2008). The gene is responsible for intrahepatic cholestasis of pregnancy 3 (ICP3, OMIM 614972). Heterozygotes variants in the *ATP8B1* gene on chromosome 18q21, except intrahepatic cholestasis of pregnancy 1 (ICP1, OMIM 147480), can also cause progressive familial intrahepatic cholestasis 1 (PFIC1, OMIM 211600) and benign recurrent intrahepatic cholestasis 1 (BRIC1, OMIM 243300).^1^ ABCC2, encoded by the ATP-Binding Cassette, Subfamily C, Member 2 (*ABCC2* gene), is an integral membrane glycoprotein expressed mainly in the canalicular (apical) membrane liver cells. It belongs to the ATP-binding cassette transporter superfamily and transports endogenous and exogenous anionic conjugates from hepatocytes to bile. The *TJP2* gene, localized on 9q21.11, encodes tight junction protein-2, which belongs to a family of membrane-associated guanylate kinase (MAGUK) homologs involved in the organization of epithelial and endothelial intercellular junctions. Its pathogenic variants have been identified in patients with progressive familial intrahepatic cholestasis-4 (PFIC4, OMIM 615878; Sambrotta et al., 2014).

Here, we provided an insight into the possible significance of the PI*Z *SERPINA1* and suggest its potential role as a risk factor for ICP. Such a relationship seems warranted, especially in light of the PI*Z disease-modifying role in liver diseases, i.e., in triggering hepatic dysfunction in cystic fibrosis (Bartlett et al., 2009; Elborn, 2016), as a disease modifier in alcoholic (AFLD) and nonalcoholic (NAFLD) fatty liver disease (Abul-Husn et al., 2018; Strnad et al., 2019), or due to its link to higher serum ALT and AST levels (Prins et al., 2017). Moreover, PI*Z carriers are over-represented among patients with end-stage liver disease, in individuals with cryptogenic cirrhosis (Graziadei et al., 1998), or patients referred for liver transplantation due to cirrhosis (Schaefer et al., 2018). There have never been any data published, nor do we provide evidence suggesting an association between PI*S polymorphic variant and clinically relevant risk of liver injury. This is in line with results published by others (Abul-Husn et al., 2018; Strnad et al., 2019).

The exact risk of liver disease in PI*MZ adults is currently unknown, and symptomatic liver dysfunction is not commonly observed in PI*ZZ individuals. Yet, an odds ratio for developing chronic liver disease by adult PI*Z heterozygotes varies between 1.8 and 3.1 (Fra et al., 2016). Clinical symptoms might be augmented by coexisting conditions, such as alcohol abuse, non-alcoholic fatty liver disease, or cystic fibrosis (*CFTR* variants; Strnad et al., 2020) as well as being significantly affected by age. PI*Z carriers become symptomatic for liver disease later in life – on average PI*ZZ at the age of 58years, PI*MZ at 73, and PI*SZ at 66years of age (Irving et al., 2014). The mechanisms behind AATD-mediated liver disease are not fully understood. The retention of Z AAT protein results in the formation of Z aggregates within the endoplasmic reticulum (ER) and contributes to hepatocyte damage as the key process, due to the fact that the aggregates have a significant gain-of-function hepatotoxic effect (Teckman, 2013; Bouchecareilh, 2020). Accordingly, the inclusion bodies (IB) are the histopathological hallmark of AATD liver disease. In a mouse model of AT deficiency-associated liver disease, the norursodeoxycholic acid (norUDCA), a side-shortened homolog of UDCA, has been shown to induce autophagy and, therefore, reduce the burden of Z AAT protein deposits (Hidvegi et al., 2010; Tang et al., 2018). Both UDCA and norUDCA are used in clinical practice to limit Z AAT induced liver damage.

Ursodeoxycholic acid is also commonly used for the treatment of ICP. Its effects are thought to occur by improving biliary flow, enhancing the protective mechanisms, and protecting the liver from bile acid-induced apoptosis (Pusl et al., 2008; Bicocca et al., 2018). Studies have demonstrated that UDCA treatment is associated with a reduction of pruritus (Kong et al., 2016). Unfortunately, clear evidence for the clinical benefit of UDCA in reducing serious pregnancy complications is not extensive. Most studies suggest the fetal stillbirth in patients with ICP is caused by the toxic influence of bile acids on fetal heart cells (Williamson et al., 2001; Ozel et al., 2020). Therefore, according to some authors, UDCA treatment may reduce the impact of the abovementioned pathological and toxic mechanisms that are implicated in the etiology of stillbirth in ICP, such as fetal arrhythmia (Miragoli et al., 2011).

## Conclusion

The prevalence of *SERPINA1* PI*Z variant in a group of women with intrahepatic cholestasis is higher compared to general population. Our data provide the first evidence for association between *SERPINA1* Z variant and ICP.

## Limitations

Given the small size of the analyzed group, these results should be interpreted with caution and further verified in ICP patients. This hypothesis should be also tested with two different control groups: pregnant women without ICP and non-pregnant women. In case this is confirmed, screening for SERPINA1 PI*Z might be used to emerge a group of women who might be more prone to ICP. It may also provide an inspiration for new treatment options or schemes dedicated to pregnant PI*Z carriers.

## Data Availability Statement

The original contributions presented in the study are included in the article/supplementary material, further inquiries can be directed to the corresponding authors.

## Ethics Statement

The studies involving human participants were reviewed and approved by Ethics Committees of Poznan University of Medical Sciences (no. 197/18). The patients/participants provided their written informed consent to participate in this study. Written informed consent was obtained from the individual(s) for the publication of any potentially identifiable images or data included in this article.

## Author Contributions

PK, MK, PG, ML, JC-W, MW, and AJ-S: conception or design of the work. PK, MK, PG, AR, JC-W, and AJ-S: data collection. PK, MK, AM, PG, ML, EW-O, AR, JC-W, MW, and AJ-S: data analysis and interpretation. PK, MW, JC-W, and AJ-S: drafting the article. MK, AM, AR, EW-O, MW, and AJ-S: critical revision of the article. All authors contributed to the article and approved the submitted version.

## Conflict of Interest

The authors declare that the research was conducted in the absence of any commercial or financial relationships that could be construed as a potential conflict of interest.

## Publisher’s Note

All claims expressed in this article are solely those of the authors and do not necessarily represent those of their affiliated organizations, or those of the publisher, the editors and the reviewers. Any product that may be evaluated in this article, or claim that may be made by its manufacturer, is not guaranteed or endorsed by the publisher.

## Footnotes

^1^ www.omim.org/entry/602397?search=ATP8B1&highlight=atp8b1

## Abbreviations

ICP: Intrahepatic cholestasis of pregnancy
AATD: Alpha-1 antitrypsin deficiency
UDCA: Ursodeoxycholic acid
GA: Gestational age
WOG: Week of gestation
ALT: Alanine aminotransferase
AST: Aspartate aminotransferase
TBA: Total bile acids
CS: Cesarean section
DBS: Dry blood spots
ER: Endoplasmic reticulum
IB: Inclusion bodies
